# Trabectedin-Associated Myocardial Infarction With Nonobstructive Coronary Arteries

**DOI:** 10.1016/j.jaccas.2026.108321

**Published:** 2026-05-14

**Authors:** Srikiran Dasari, Eduardo Alfredo Aviles, Brijesh Patel

**Affiliations:** a Division of Internal Medicine, Indiana University School of Medicine, Indianapolis, Indiana, USA; b Division of Cardiovascular Medicine, Indiana University School of Medicine, Indianapolis, Indiana, USA

**Keywords:** acute coronary syndrome, myocardial infarction, treatment

## Abstract

**Background:**

Trabectedin is a marine-derived alkylating agent used to treat soft tissue sarcomas.

**Case Summary:**

We present a case of myocardial infarction with nonobstructive coronary arteries (MINOCA) in a 58-year-old man with no prior cardiac history who was receiving trabectedin for metastatic liposarcoma. Initial work-up revealed elevated high-sensitivity troponin, unremarkable electrocardiogram and echocardiogram, and mild nonobstructive coronary disease on coronary angiography. The patient's chest discomfort recurred days after another chemotherapy cycle, and it was hypothesized that coronary vasospasm produced his symptoms. Prophylactic amlodipine and isosorbide mononitrate were administered around subsequent cycles, with complete resolution of symptoms.

**Discussion:**

Recurrent episodes of acute coronary syndrome after trabectedin infusions that resolved with vasodilator pretreatment supported trabectedin-induced coronary vasospasm as the mechanism of MINOCA in this patient. This is to our knowledge the first reported such case with trabectedin.

**Take-Home Message:**

Patients who develop trabectedin-associated MINOCA may benefit from pretreatment with anti-vasospastic antianginals to allow for safe continuation of therapy.

## Visual Summary

**Figure:**
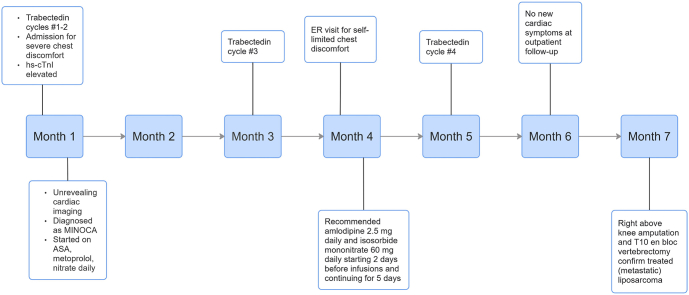

**Visual Summary:**
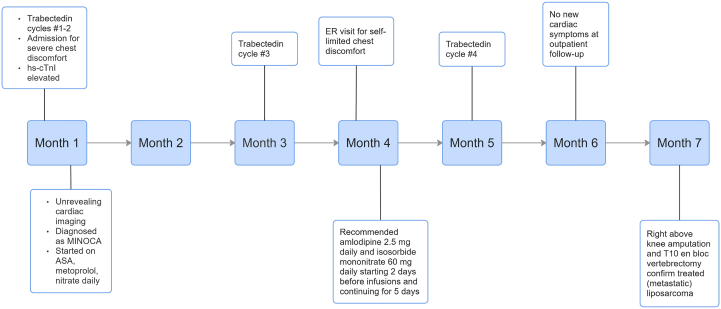
Timeline of Case Presentation Seven-month outline of clinical case. The patient developed severe, exertional, radiating chest discomfort 5 days after cycle 2 of trabectedin which prompted medical evaluation. High-sensitivity troponin was elevated, but coronary angiogram was largely unrevealing. He was discharged with metoprolol succinate and isosorbide mononitrate. About 2 months later, he developed recurrent episodes of chest discomfort 2 days after cycle 3, which were self-limited. Following suspicion of MINOCA due to coronary vasospasm, he was initiated on a regimen of prophylactic amlodipine and isosorbide mononitrate for 1 week starting a few days before subsequent chemotherapy sessions. He successfully completed the course of neoadjuvant chemotherapy and developed no further cardiac symptoms after administration of cycle 4. ASA = aspirin; ER = emergency room; hs-cTnI = high-sensitivity cardiac troponin I; MINOCA = myocardial infarction with nonobstructive coronary arteries.

Trabectedin is a marine-derived alkylating chemotherapy agent that forms adducts in the minor groove of DNA by binding to guanine residues, resulting in strand breaks, cell cycle arrest, and apoptosis.^1^ This drug is approved by the U.S. Food & Drug Administration to treat soft tissue sarcomas, specifically unresectable or metastatic liposarcoma or leiomyosarcoma, in patients who have failed anthracycline therapy.^2^ Nausea, vomiting, and fatigue are common side effects of trabectedin, although more serious adverse drug reactions such as hepatotoxicity, rhabdomyolysis, and cardiotoxicity have occurred. Here, we describe a case of myocardial infarction with nonobstructive coronary arteries (MINOCA) due to suspected trabectedin-induced coronary vasospasm in a patient with metastatic liposarcoma.

**Take-Home Messages**

• Trabectedin may cause coronary vasospasm resulting in MINOCA even in patients without significant cardiac history.
• Early recognition of this cardiovascular adverse event is clinically relevant, because pretreatment with calcium-channel blockers and nitrates may prevent recurrence and allow for safe continuation of trabectedin in patients with metastatic liposarcoma who have failed previous chemotherapy agents.

## History of Presentation

A 58-year-old man presented with symptoms of chest discomfort radiating to the jaw and left shoulder, dyspnea, cough, leg swelling, and diaphoresis lasting 14 hours. His symptoms began while attempting to have a bowel movement, and they increased in severity with ambulation, prompting the emergency room visit. He self-administered 6 aspirin tablets (81 mg) before coming to the hospital. His vital signs were stable, and physical examination revealed 1+ bilateral lower extremity edema to the shins.

## Past Medical History

The patient's medical history included right leg myxoid liposarcoma metastatic to the T10 vertebra on trabectedin every 3 weeks (second cycle 5 days before presentation), obesity class III, obstructive sleep apnea on nocturnal continuous positive airway pressure, hypothyroidism, pulmonary sarcoidosis, and tobacco use. His first cycle of trabectedin was uncomplicated.

## Investigations

**Figure 1:**
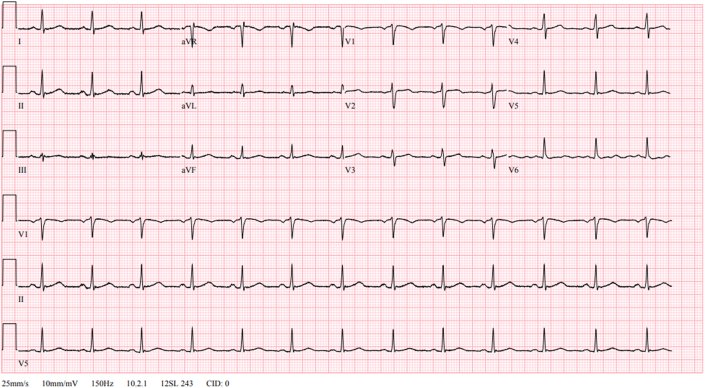
Initial Electrocardiogram With Normal Sinus Rhythm Electrocardiogram demonstrates a ventricular rate of 78 beats/min, P-R interval of 158 ms, QRS duration of 88 ms, QTcB of 465 ms, and no ST-segment changes.

**Figure 2:**
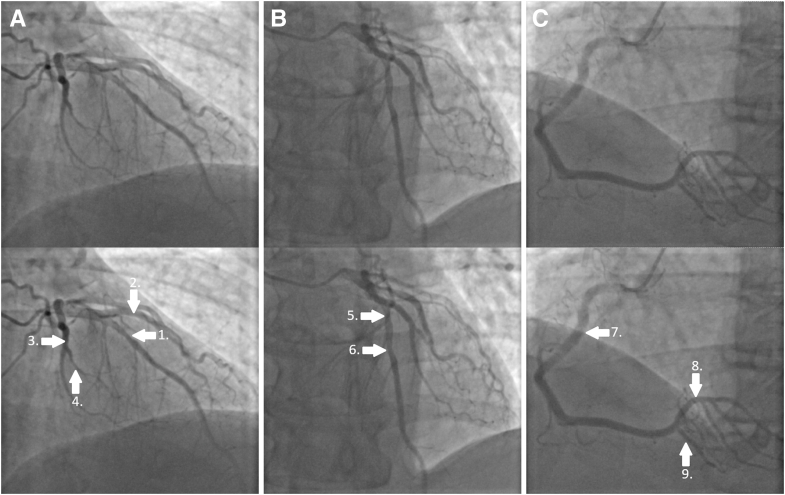
Coronary Angiogram With Mild Nonobstructive Coronary Disease of the Left Anterior Descending Artery (A) RAO cranial view: 1) large-caliber left anterior descending artery with TIMI flow grade 3 and a mild luminal irregularity denoted by the arrow between the 2 septal perforator branches; 2) patent diagonal branch; 3) large-caliber left circumflex artery which is angiographically normal with TIMI flow grade 3; and 4) patent obtuse marginal branch. (B) LAO cranial view: 5 and 6) mild luminal irregularities of the left anterior descending artery. (C) LAO view of right coronary artery: 7) Large dominant right coronary artery which is angiographically normal with TIMI flow grade 3; 8) posterior left ventricular branch; and 9) posterior descending artery. LAO = left anterior oblique; RAO = right anterior oblique.

Pertinent laboratory data included peak high-sensitivity cardiac troponin I (hs-cTnI) 593 ng/L, B-type natriuretic peptide 233 pg/mL, and D-dimer 749 ng/mL. Electrocardiogram (ECG) demonstrated normal sinus rhythm (Figure 1). Transthoracic echocardiography showed an ejection fraction of 65%, mild left atrial dilation, normal wall motion, and grossly normal valves. Chest computed tomography pulmonary embolism protocol was without acute findings. The patient was started on a heparin drip and sent to the cardiac catheterization laboratory. Coronary angiography revealed mild nonobstructive coronary disease with mild luminal irregularities of the left anterior descending artery (Figure 2) and a left ventricular end-diastolic pressure of 20 mm Hg.

## Differential Diagnosis

Given symptom characteristics (eg, radiating chest discomfort, dyspnea), recent initiation of trabectedin, and troponin elevation with lack of significant electrocardiographic and angiographic findings, the 2 prioritized differential diagnoses included MINOCA and myocardial injury.

## Management

The patient was discharged the next day with aspirin 81 mg daily, metoprolol succinate 12.5 mg daily for possible microvascular disease, and isosorbide mononitrate 30 mg daily. He elected to receive his third cycle of chemotherapy 2 months later, and he subsequently developed chest pressure and jaw numbness, waking him up from sleep 2 days after the infusion. His pain initially responded to sublingual nitroglycerin but returned a few hours later, prompting another emergency room visit. The patient was asymptomatic on arrival but reported a few similar episodes after cycle 3 of trabectedin. Repeat laboratory results included hs-cTnI 21 ng/L and D-dimer 678 ng/mL in the setting of active cancer. Repeat ECG and chest computed tomography were again normal, and he was discharged home.

He was evaluated by cardio-oncology 5 days later as an outpatient. In addition to continuing his home metoprolol, he was started on a protocol consisting of amlodipine 2.5 mg and increased-dose isosorbide mononitrate 60 mg daily, both beginning 2 days before subsequent chemotherapy cycles and continuing for an additional 5 days (total, 1 week). His aspirin was also stopped.

## Outcome and Follow-Up

The patient underwent cycle 4 of trabectedin 2 months later. He denied further episodes of chest discomfort or other concerning cardiac symptoms at any point after this cycle on evaluation at his oncology appointment 1 month later.

He underwent right above-the-knee amputation and T10 vertebrectomy within the next few months. Histopathologic diagnosis was consistent with treated myxoid liposarcoma.

## Discussion

The overall risk of cardiovascular adverse events (CAEs) associated with trabectedin is generally low and ranges from rate and rhythm disturbances (eg, sinus tachycardia, atrial arrhythmias) to more serious complications such as acute heart failure exacerbation, myocardial infarction, and cardiac arrest.^3^^,^^4^ Early phase I-II clinical trials demonstrated a CAE rate of 1.4% to 1.8% (mostly arrhythmias, tachycardia, and palpitations), although a recent systematic review estimated a higher rate of 3.4%, with heart failure occurring as frequently as arrhythmias and ECG abnormalities.^3^^,^^4^ The risk of CAEs is greater in patients who have prior cardiovascular history, use cardiac medications, and are older than 65.^5^ One case reported fulminant myocardial injury a few days after trabectedin with high peak hs-cTnI level (37,993 ng/L), monomorphic ventricular tachycardia, and biopsy-confirmed, drug-induced myocardial toxicity with the presence of cytoplasmic vacuoles, although this occurred in the setting of multiorgan failure.^6^ Previous anthracycline exposure may also increase the risk of trabectedin-associated CAEs because of their cardiotoxicity via topoisomerase 2β inhibition and reactive oxygen species generation.^7^ Given that many patients start trabectedin after treatment failure with anthracyclines, one must remain vigilant for CAEs in patients receiving trabectedin, including those without significant prior cardiac history.

**Figure 3:**
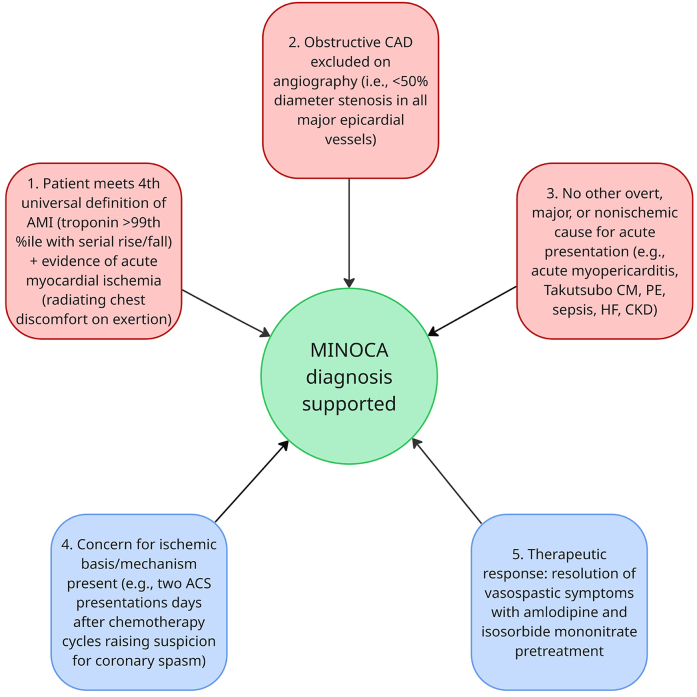
Clinical Findings Supporting a Diagnosis of MINOCA Points 1 to 3 summarize key diagnostic criteria for MINOCA from the American College of Cardiology/American Heart Association guidelines. Causes of myocardial injury that are nonischemic cardiac (eg, acute myopericarditis, Takutsubo cardiomyopathy) and nonischemic extracardiac (eg, pulmonary embolism, sepsis) should be excluded. Points 4 and 5 describe specific features of this case that increased the suspicion for coronary vasospasm. ACS = acute coronary syndrome; AMI = acute myocardial infarction; CAD = coronary artery disease; CM = cardiomyopathy; CKD = chronic kidney disease; HF = heart failure; MINOCA = myocardial infarction with nonobstructive coronary arteries; PE = pulmonary embolism.

This was a case of neoadjuvant-intent trabectedin in which the patient achieved treatment response to both primary and metastatic lesions after 4 cycles, however cycles 2 and 3 were complicated by episodes of radiating chest pressure, the first of which was diagnosed as MINOCA (Figure 3). These episodes were preceded by trabectedin infusion 2 to 5 days beforehand. Given the symptom timing and quality, normal ECG with significant troponin elevation, mild nonobstructive coronary disease on coronary angiography, and complete resolution of symptoms with amlodipine and isosorbide mononitrate pretreatment, his MINOCA was likely was due to trabectedin-induced coronary vasospasm, to our knowledge the first reported case related to trabectedin in the literature. The patient met criteria for the fourth universal definition of myocardial infarction after cycle 2, and he appeared to experience recurrent vasospastic episodes after cycle 3, which did not warrant hospital admission owing to spontaneous resolution.

The pathophysiology of MINOCA is heterogeneous and includes both atherosclerotic causes (eg, plaque disruption) as well as nonatherosclerotic and mixed etiologies (eg, epicardial coronary vasospasm, coronary microvascular dysfunction, coronary artery thromboembolism, spontaneous coronary artery dissection).^8^ Diagnostic work-up varies by cause (eg, angiography and intravascular imaging for plaque disruption and coronary embolism, functional assessment for microvascular dysfunction, and symptom modulation with coronary vasodilators for epicardial spasm).^8^ A recent Bayesian meta-analysis investigated medications for secondary prevention in over 12,000 MINOCA patients, reporting significantly decreased all-cause mortality with statins (hazard ratio: 0.61, Bayes factor: 32.20) and significantly decreased major CAEs with renin-angiotensin-aldosterone system inhibitors (hazard ratio: 0.74, Bayes factor: 8.98) but neutral prognostic evidence for beta-blockers and dual antiplatelet therapy.^9^ Table 1 outlines the role of various pharmacotherapies in MINOCA.

**Table 1:** Role of Therapeutic Strategies in MINOCA Based on Current Evidence

Medication/Pharmacotherapy	Role in MINOCA
Aspirin ± DAPT	Reduce platelet aggregation, aspirin reasonable if any atherosclerotic plaque present, aspirin used if plaque disruption or SCAD are confirmed as the etiology although limited role in isolated vasospasm,^8^ DAPT provided neutral prognostic benefit in MACE and all-cause mortality in a contemporary Bayesian meta-analysis of >12,000 MINOCA patients.^9^
ACEi/ARB	Inhibit RAAS, potential benefit with endothelial dysfunction and vascular remodeling, clinical benefit in the presence of reduced ejection fraction or left ventricular dysfunction,^8^ neutral prognostic benefit in MINOCA in a recent meta-analysis.^9^
Beta-blocker	Reduce myocardial oxygen demand, beneficial in the setting of proven coronary microvascular dysfunction but may exacerbate coronary vasospasm, also indicated for post-MI left ventricular dysfunction and SCAD.^9^^,^^10^
CCB	Inhibit vascular smooth muscle contraction, first-line/cornerstone therapy for INOCA and MINOCA due to epicardial coronary vasospasm, may also alleviate symptoms of coronary microvascular dysfunction.^9^^,^^10^
Nitrate	Produce nitric oxide–mediated vasodilation, short-acting sublingual and intracoronary routes of administration beneficial for acute symptoms of coronary vasospasm, long-acting nitrates beneficial in INOCA and MINOCA due to coronary spasm although nitrate tolerance must be considered, nicorandil may be used third-line for spasm.^9^^,^^10^
Statin	Promote plaque stabilization and reduce endothelial dysfunction, recommended if any atherosclerotic plaque or endothelial impairment present, strong benefit for secondary prevention in the current literature after MINOCA due to coronary vasospasm, plaque disruption (use high-intensity), and coronary embolism^8^^,^^9^

Adapted from American College of Cardiology/American Heart Association recommendations^8^^,^^10^ with inclusion of data from a contemporary Bayesian meta-analysis.^9^ CCBs provide clinical benefit in MINOCA secondary to both epicardial coronary vasospasm (first-line) as well as coronary microvascular dysfunction. Short- and long-acting nitrates are beneficial for coronary spasm, although nitrate tolerance is a potential complication with prolonged use. Statins are indicated in multiple etiologies of MINOCA including coronary vasospasm, plaque disruption, and coronary embolism, and they may be associated with decreased all-cause mortality in such patients. Beta-blockers are suitable in the setting of coronary microvascular dysfunction and SCAD although they may exacerbate coronary vasospasm. Aspirin is employed when plaque disruption or SCAD are the cause of MINOCA, although DAPT may not yield prognostic benefit. As with beta-blockers, RAAS inhibitors are indicated with post-MI left ventricular dysfunction but may not yield prognostic benefit in MINOCA.

ACEi = angiotensin-converting enzyme inhibitors; ARB = angiotensin II receptor blockers; CCB = calcium-channel blocker; DAPT = dual antiplatelet therapy; INOCA = ischemia with nonobstructive coronary arteries; MACE = major adverse cardiovascular events; MI = myocardial infarction; MINOCA = myocardial infarction with nonobstructive coronary arteries; RAAS = renin-angiotensin-aldosterone system; SCAD = spontaneous coronary artery dissection.

The 2023 American College of Cardiology/American Heart Association guidelines recommend calcium-channel blockers (CCBs) and long-acting nitrates as first- and second-line therapy, respectively, for vasospastic angina due to epicardial spasm.^10^ In contrast to microvascular angina, where beta-blockers are the first-line therapy, vasospastic angina can worsen with beta-blocker exposure owing to unopposed α-receptor activity. Patients receiving trabectedin who develop chest discomfort or MINOCA may benefit from this unique pretreatment strategy of CCBs and long-acting nitrate medications starting a few days before chemotherapy infusions to prevent recurrent vasospastic complications.^10^ This approach may allow patients who have failed anthracycline therapy previously to continue trabectedin when they otherwise may have not been able to.

Amlodipine was selected as the CCB component of this regimen because its duration and half-life are longer than those of other drugs in this class. Whereas amlodipine has a terminal elimination half-life of 30 to 50 days and reaches steady-state plasma levels in 7 to 8 days of once-daily dosing, nifedipine's elimination half-life is only 7 hours for the extended-release tablet and a mere 2 hours for the immediate-release capsule, increasing the need for multiple daily doses.^2^ The above regimen of amlodipine 2.5 mg and isosorbide mononitrate 60 mg may be modified on a case-by-case basis depending on patient symptoms and therapeutic response. For example, these medications can be uptitrated near infusions (eg, 10 and 120 mg, respectively) to achieve adequate symptom control. Hence, nifedipine may be used as an alternative for coronary spasm in this clinical setting.

The timing of ischemic symptoms in relation to trabectedin administration highlights the need for greater clinical awareness of delayed vasospastic events, particularly within the first week after infusion. As provocative testing and advanced cardiac imaging are not routinely performed during the acute presentation, careful attention to symptom timing and reproducibility across treatment cycles may provide important clues to a vasospastic mechanism and help identify patients who would benefit from closer monitoring or early referral for coronary functional assessment.

## Conclusions

This report describes a unique case of trabectedin-associated MINOCA due to suspected coronary vasospasm. Repeat events were successfully mitigated with amlodipine and isosorbide mononitrate pretreatment. Symptom resolution after the addition of nitrate and non-nitrate therapies indicated for vasospastic angina supported epicardial spasm as the etiology of MINOCA in this case. The mechanisms of acute cardiac myocyte injury with trabectedin warrant further study.

## Funding Support and Author Disclosures

The authors have reported that they have no relationships relevant to the contents of this paper to disclose.
